# Functional connectivity of brain networks with three monochromatic wavelengths: a pilot study using resting-state functional magnetic resonance imaging

**DOI:** 10.1038/s41598-022-20668-9

**Published:** 2022-09-28

**Authors:** Marc Argilés, Bernat Sunyer-Grau, Sílvia Arteche-Fernandez, Cleofé Peña-Gómez

**Affiliations:** 1 School of Optics and Optometry, Universitat Politècnica de Catalunya, Terrassa, Catalonia Spain; 2 BarcelonaBeta Brain Research Center (BBRC), Pasqual Maragall Foundation, Barcelona, Catalonia, Spain

**Keywords:** Cognitive neuroscience, Medical research

## Abstract

Exposure to certain monochromatic wavelengths can affect non-visual brain regions. Growing research indicates that exposure to light can have a positive impact on health-related problems such as spring asthenia, circadian rhythm disruption, and even bipolar disorders and Alzheimer’s. However, the extent and location of changes in brain areas caused by exposure to monochromatic light remain largely unknown. This pilot study (N = 7) using resting-state functional magnetic resonance shows light-dependent functional connectivity patterns on brain networks. We demonstrated that 1 min of blue, green, or red light exposure modifies the functional connectivity (FC) of a broad range of visual and non-visual brain regions. Largely, we observed: (i) a global decrease in FC in all the networks but the salience network after blue light exposure, (ii) a global increase in FC after green light exposure, particularly noticeable in the left hemisphere, and (iii) a decrease in FC on attentional networks coupled with a FC increase in the default mode network after red light exposure. Each one of the FC patterns appears to be best arranged to perform better on tasks associated with specific cognitive domains. Results can be relevant for future research on the impact of light stimulation on brain function and in a variety of health disciplines.

## Introduction

Exposure to light in humans has numerous implications beyond visual perception. Recently, multiple potential uses of light have been described for the treatment of varying clinical conditions^1^. Exposure to wavelengths between 460 and 470 nm can modify circadian rhythms^2^. Also, blue light (~ 450 nm) has been used in multiple clinical conditions such as in night shifts workers to improve their sleep–wake cycles^3^, in spring asthenia and bipolar disorder^4^, and depression in pregnant women^5^.

In all these cases the wavelengths used were around 450 nm, the wavelength to which melanopsin, the light-sensitive pigment present in intrinsically photosensitive retinal ganglion cells (ipRGC), is most sensitive^6^. These cells constitute a non-visual pathway that connects the human eye with the suprachiasmatic nucleus in the hypothalamus^7^, the master circadian pacemaker responsible for controlling the timing of the sleep–wake cycle and regulating circadian rhythms, in other brain areas and throughout the body^8^.

Other studies have shown that exposure to LED light before bedtime, which is rich in the blue range of wavelengths^9^, can influence sleep patterns^10^. Furthermore, night-time blue-light exposure has been associated with health problems such as breast and prostate cancers^11^. In this regard, certain wavelengths like blue-enriched light near 440 nm have been described to mighty modulate cognitive abilities such as attention and performance in cognitive tasks^12^. Other studies have shown the efficacy of specific light interventions to reduce daytime sleepiness^13^, depressive symptoms^14^ improve motor skills in brain injury^15^, and improving visuospatial attention^16^.

Interestingly, other wavelengths different from blue light have also been demonstrated to have non-visual effects. For example, wavelengths corresponding to green light have been shown to ameliorate the symptoms of migraine^17^. In animal models, green light has been shown to provide beneficial antinociceptive effects^18^. Likewise, red light has been reported to increase alertness without undesired modifications in circadian rhythms^19^.

On the other hand, several studies have reported that exposure to different wavelengths over varying periods while performing cognitive tasks, results in increased activity in specific brain regions. In an auditory “2-back” working memory task increased brain activity was found using functional magnetic resonance imaging (fMRI) in the middle frontal gyrus and the supramarginal gyrus with blue light (470 nm), compared with green light (550 nm)^20^. In the visual system, one study found increased activation in posterior and occipital regions using red-light stimulation in esotropia and blue light in exotropia^21^. Interestingly, other authors found that melatonin suppression by blue (470 nm) and amber (590 nm) light exposure is hemispherically dependent in the retina, observing more melatonin suppression in the nasal hemifield^22^.

Overall, there is a large amount of scientific evidence showing potential clinical uses for visible light in the treatment of multiple health problems. However, the underpinning neural mechanisms, the brain areas involved, and how they are functionally arranged is less known. The examination of brain functional network connectivity under-resting state fMRI (rs-fMRI) conditions enables the understanding of how different brain areas can be functionally re-organized after an external intervention^23^.

In this rs-fMRI functional connectivity (FC) pilot study, we aim to identify the brain regions affected by measuring the whole-brain FC before and after one minute of passive light exposure to three different monochromatic lights: blue, green and red. To the best of our knowledge, this is the first study to observe and compare the possible brain connectivity changes seen after exposure to different monochromatic wavelengths. Therefore, we used the rs-fMRI that enables a neat evaluation of the changes on the FC organization.

## Results

**Figure 1:**
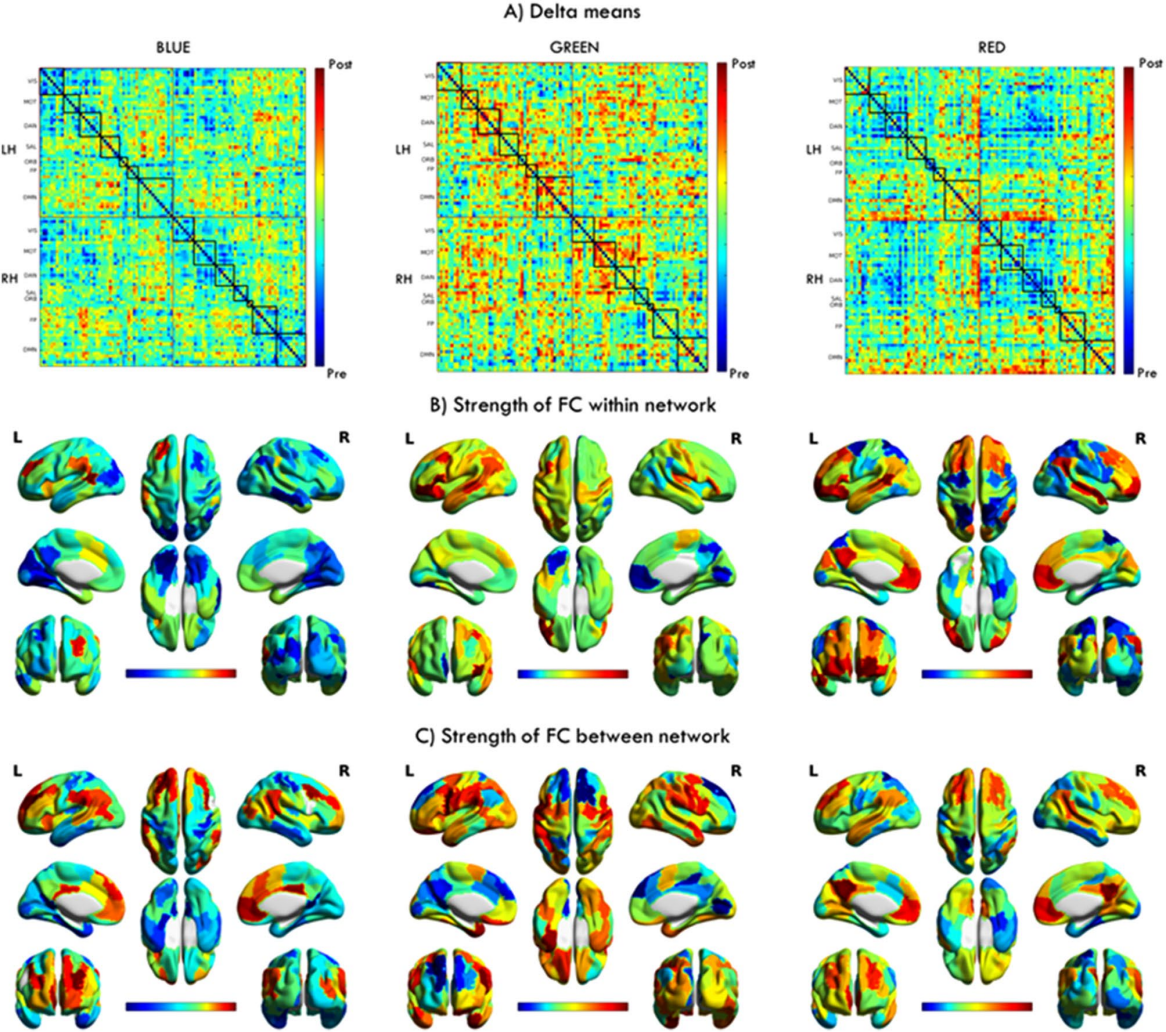
In panel A are shown in matrix form the average differences (delta) across subjects between post- and pre-stimulation for each kind of light exposure. The within network connectivity for each RSN corresponds to the black squares inside the matrices, while the between network connectivity are the values outside the black squares. In panel B and C are projected the strength of FC corresponding to the within and between connectivity, respectively. The color bar in the figure indicates the directionality of the difference in FC. Red painted areas indicate that FC was higher, and blue areas show that FC was lower, after light exposure compared to before. All the matrices in panel A follow the same structure: LH: Left hemisphere, RH: Right hemisphere, VIS: visual network, ASM: auditory and somatosensory network, DAN: the dorsal attention network, SAL: saliency network, ORB: orbitofrontal-temporopolar network, FP: frontoparietal, DMN: default mode network. Color bars indicate the normalized strength of FC change ranging from − 3 to + 3.

The general characterization of the effects produced by light stimulation is shown in Fig. 1. Red/blue color indicates that FC was higher after/before the light stimulation, respectively. We computed FC strength within networks and also between networks for each type of light exposure. The within network values for each RSN for each hemisphere are delimitated by a black square inserted in the delta matrix (Fig. 1A). Therefore, the between network connectivity values are outside from the black squares. An ANOVA of repeated measures was conducted for the comparisons between monochromatic lights.

### Strength of functional connectivity within networks

In Fig. 1 panel B) is shown the strength of FC within the network for each kind of light stimulation.

After blue light exposure, we observe an average decrement within network FC in the VIS network in both hemispheres, but particularly on the left (Z = − 1.44) hemisphere compared to the right (Z = − 0.77). Some regions of the left visual cortex experimented a clear decrement: in the occipital fusiform (Z = − 2.46, *P* = 0.01, MNI = − 26, − 77, − 14; Z = − 2.17, MNI = − 17, − 60, − 7, *P* = 0.03) and the cuneus (Z = − 2.23, MNI = − 6, − 82, 26, *P* = 0.02). On the other hand, the left SAL network showed an increase in FC (Z = 1.39). Particularly, in the frontal pole (Z = 2.48, MNI = − 30, 44, 30, *P* = 0.01) and in the parietal operculum (Z = 1.97, MNI = − 59, − 38, 29, *P* = 0.04). The DMN did not exhibit a clear tendency as a whole (Z_LH_ = 0.29, Z_RH_ = − 0.58). Some regions within the DMN exhibited a remarkable change, the angular gyrus showed a considerable increase (Z = 2.91, MNI = − 57, − 50, 12, *P* = 0.003), whereas the right middle temporal gyrus (Z = − 2.38, MNI = 62, − 23, − 19, *P* = 0.01) a clear disminution in FC.

After green light exposure, the left DMN (Z = 1.18) and the right MOT (Z = 1.21) networks exhibited an increment in average FC strength, but not in the right DMN (Z = − 0.28). The highest increases were seen in regions within the DMN, such as the left middle temporal region (Z = 2.19, MNI = − 58, − 32, − 1, P = 0.02), but specifically some regions of the frontal orbital cortex (Z = 2.37, MNI = − 35, 21, − 11, *P* = 0.01), inferior frontal gyrus (Z = 3.50, MNI = − 47, 33, − 3, *P* < 0.001) and middle frontal gyrus (Z = 2.60, MNI = − 41, 14, 48, *P* < 0.01). An insignificant disminution in FC was seen in the right intra-calcarine cortex (Z = − 1.84, MNI = 8, − 76, 5, *P* = 0.06) as well as in the left occipital fusiform visual cortex (Z = − 1.89, MNI = − 26, − 77, − 14, *P* = 0.05).

After red light exposure, the DAN showed a clear FC decrement bilaterally (Z_LH_ = − 1.19 and Z_RH_ = − 1.35) while the DMN exhibited a clear FC increment in the left (Z = 1.23) and a more moderate increase in the right DMN (Z = 0.88). Particularly, the highest decrease in the DAN occurred in the left superior parietal cortex (Z = − 1.99, MNI = − 22, − 51, 66, *P* = 0.04) and in the right superior parietal cortex (Z = − 2, 13, MNI = 14, -52, 66, *P* = 0.03). The highest increases in FC where in the DMN, in the angular gyrus (Z = 2.16, MNI = − 57, − 50, 12, *P* = 0.03), the left frontal pole (Z = 2.22, MNI = − 24, 61, − 1, *P* = 0.02) and in the right temporal pole (Z = 2.17, MNI = 51, 7, − 18, *P* = 0.03).

### Strength of functional connectivity between networks

In Fig. 1 panel C) is shown the strength of FC between networks for each kind of light stimulation.

After blue light exposure there was not a clear tendency of increase nor decrease in FC between any of the networks. However, there was a global decrement between whole-brain connectivity (Z = − 0.06). Specific areas of the visual network experienced a FC decrease (Z = − 2.33, MNI = 26, − 34, − 17, *P* = 0.01) as well as in the DMN, particularly the PPC (Z_LH_ = − 2.34, MNI = − 11, − 56, 13, *P* = 0.01; Z_RH_ = − 2.65, MNI = 12, − 54, 14, *P* = 0.008). In contrast, regions in the DLPFC within the SAL network (Z = 1.77, MNI = − 30, 44, 30) experienced an increase in FC but not significant.

After green light exposure, on average there was not a predominance of a specific network in FC increments or decrements. However, there was a global increment in between whole-brain connectivity (Z = 0.11). The increments were concentrated in the precentral region within the DAN (Z = 2, 13, MNI = − 48, 6, 28, *p* = 0.03) and in the temporal region (Z = 1.94, MNI = 32, 2, − 37, *P* = 0.05). Also, some decrements were present in the superior frontal cortex (Z = − 1.94, MNI = 26, 24, 50, *P* = 0.05).

After red light exposure there was no specific network clearly increasing or decreasing. On average, connectivity between networks decreased globally (Z = − 0.11). The DMN showed an increased FC, particularly noticeable on the left and right PPC (Z_LH_ = 2.82, MNI = − 6, − 53, 33, *P* = 0.004; Z_LH_ = 2.81, MNI = 7, − 52, 31, *P* = 0.004). On the other hand, the DAN, particularly the parietal regions, showed a decreased FC (Z = − 2.22, MNI = − 22, − 51, 66, *P* = 0.02).

After performing the ANOVA repeated measures, a total of 92 connections were found to have a statistically significant change (*p* < 0.05) as a function of light stimulation. However, after FDR corrected for multiple comparisons, a total of 4950 (one for each connection), no significant *p* values were surviving this correction. Here we report those connections that were significant at uncorrected *p* value < 0.01 (a total of 46).

**Figure 2:**
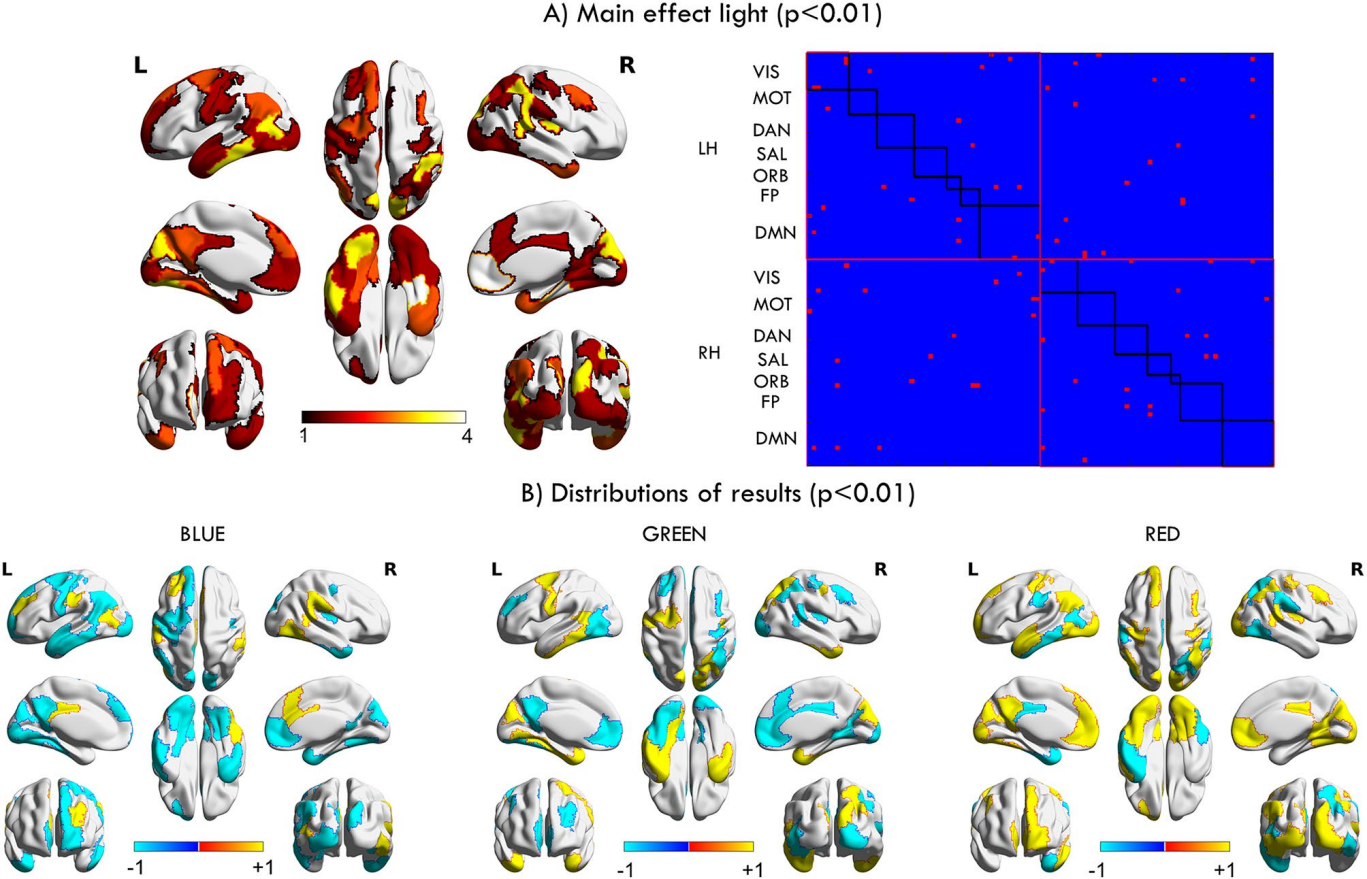
Projections that were significant at uncorrected *p* value < 0.01 into the brain surface. In panel (**A**) on the left is shown the projection onto the brain of the main effect of light. To note that any given ROI can have a maximum of 99 significant connections with the rest of the ROIs. This can be observed on the right side of panel (**A**), where each row (and column) in the symmetrical binary matrix corresponds to a ROI, and a red square inside of the matrix represents a significant connection (each connection involves two ROIs). Therefore, adding together the values across the rows yielded a value for each ROI indicating the most sensitive areas after light exposure which is projected onto the brain surface in the left side of panel A. The heat map illustrates all the areas sensitive to light exposure, being the yellowish the most sensitive and the red-dark the less. The colorbar illustrates the number of times that appeared in the left matrix. In panel (**B**), are the brain projections associated with each kind of light exposure, after that for the connections described in panel A) we assessed the highest/lowest value and their correspondence to the type of light exposure. In panel (**B**), hot colors indicate the values where the ROIs are increasing the FC, while blue colors indicate the opposite. Abbreviations: LH: Left hemisphere, RH: Right hemisphere, VIS: visual network, ASM: auditory and somatosensory network, DAN: the dorsal attention network, SAL: saliency network, ORB: orbitofrontal-temporopolar network, FP: frontoparietal, DMN: default mode network.

Those connections with *p* < 0.01 were projected into the brain. Figure 2 in panel A are shown the significant connections and their projection into the brain. It is important to note that each connection involves two ROIs, and therefore one region can be present more than once and this is captured by the heat map. Particularly, we observe that MPFC, visual areas and ventral medial temporal areas exhibit a higher main effect of light. At network level, we observed the DMN mainly in the left hemisphere and the VIS in both hemispheres. Also, some areas involve other networks such as the FEF corresponding to the DAN, the ventral medial temporal areas corresponding to the ORB, and the right inferior parietal corresponding to the SAL network.

For each one of these connections, we examined under which of the three stimulation conditions the connection was the highest or the lowest. Projections into the brain surface of this computation are shown in Fig. 2 panel B). Compared with the other two, blue light stimulation indicated a general decrease of FC in these significant areas, except in those areas associated with the salience network such as the anterior cingulate cortex and the supramarginal gyrus (F = 7.91, *P* = 0.007). Particularly, within the VIS regions (F = 12.77, *P* = 0.001), in several regions of the DMN such as the left parietal with the temporal pole (F = 15.47, *P* < 0.001), and in the left PFC with the temporal pole (F = 9.24, *P* = 0.004), showed a decreased FC in comparison with the other two groups.

The green light exposure compared with the other two light exposures indicated a decrement in the FC integrity in the DMN particularly evident in the midline, as seen in connections between the PPC and the somatomotor areas (F = 7.97, *P* = 0.007) and between the PFC and visual regions (F = 11.7, *P* = 0.001), however, in the lateral DMN there was a clear FC increment, for example between the parietal DMN regions and the temporal pole (F = 15.47, *P* < 0.001). Also, other regions belonging to the SAL network experienced a higher FC such as the superior parietal cortex with the DLPFC (F = 7.43, *P* = 0.009) and portions of the supramarginal gyrus with the DLPFC, (F = 18.57, *P* < 0.001). Regarding the VIS cortices, we did not observe a clear effect of increase or decrease in FC of the whole visual cortex. Thus, we observed a higher FC in the midline of the occipital cortex intrinsically (F = 7.83, *P* = 0.007) as well as connections with somatomotor regions (F = 10.31, *p* = 0.002), whereas in the left lateral occipital (F = 9.41, *P* = 0.004) and left fusiform area (F = 9.96, *P* = 0.003) a decrement of FC with the MPFC was observed in comparison with the other two groups.

After the red-light exposure, the connections belonging to the DMN such as the PFC with the PPC (F = 8.55, *P* = 0.005) showed an increased FC, suggesting a higher integrity within the DMN. Also, the PFC and VIS showed an increased FC (F = 12.77, *P* = 0.001). In contrast, parietal regions associated with attentional networks such as the supramarginal gyrus (F = 7.81, *P* = 0.007), and superior parietal areas (F = 9.48, *P* = 0.004) showed a decreased FC.

## Discussion

In this pilot rs-fMRI study carried out in a young, small sample, age = 28.00 ± 4.50 years (range 21–33 years), we found that 1 min of passive stimulation of red (635 nm), green (516 nm) and blue (464 nm) monochromatic lights resulted in changes in functional brain network connectivity patterns.

Interestingly, these FC changes were occurring beyond the visual regions, a FC reorganization was observed in different brain networks. Furthermore, the reorganized patterns of FC were dependent on the type of light exposure. In general, after red light exposure, we observed a decrease within network connectivity in the attentional networks coupled with an increase in FC in the DMN. After green light stimulation, FC was reduced in the medial DMN and enhanced in the DAN. And finally, after blue light exposure, we observed a reduction in within network connectivity on all the RSNs but the SAL.

After the red light exposure, within network connectivity in the DAN and the SAL decreased, whereas the FC within and between the DMN exhibited an increase. Thus, red light exposure seems to disrupt regions associated with goal-oriented tasks such as working memory while enhancing cortical regions associated with memory recall or mind-wandering such as the DMN^28^. DMN has been related to an improvement of cognitive control^29^, an improvement from two-month of meditation^30^, and it has been reported to have decreased functional connectivity in glial tumor patients^31^, Parkinson’s disease^32^, unilateral amblyopia^33^ and depressive disorder^34^, although it seems that medication for major depressive disorder does not influence the organization of DMN^35^. Also, working-memory performance has been related to the activation and deactivation of the DMN^36^. Overall, our findings may suggest that red light stimulation is reorganizing the whole-brain FC in such a way that could be detrimental to the executive function but beneficial for memory-related tasks. Thus, the red light enhances this traditional dichotomic view of task-positive and task-negative performance^37^.

On the other hand, the VIS network exhibits a moderate increase in FC which might suggest that visual tasks could be performed easily. However, given that most tasks require regions that belong to the SAL and DAN networks, it is not clear what would be the performance in a visually oriented task. It is known that the DAN is activated by visual-spatial attention tasks^38^, connected to the ventral attention network^39^ and related to visual attention modulated from the ocular dominance^40^. Also, it has been shown that DAN FC changes after acute stress exposure^41^ and in sleep problems^42^. In agreement with other studies^21^, the visual cortex seems to increase its FC with red light stimulation, compared with green and blue lights.

After green light exposure, the FC integrity of the DMN is reduced, particularly in the lateral areas with the midline areas. The key core regions of the DMN such as the PPC have decreased their FC strength. In contrast, regions associated with the DAN such as the frontal eye field (FEF) or the intraparietal sulcus (IPS) have strengthened their internal connectivity as well as the connections with the rest of the cortex. FEF shows a desynchronization associated with the control of visuospatial attention tasks^43^ and it is known that it is involved in the control of ocular saccades^44^ and pursuits^45^. It has also been observed a decreased functional activity between the right frontal eye field in social network users^46^ and cases of amblyopia in adults^47^ and strabismus^48^. Interestingly, we find an opposite effect of the green light from the red-light stimulation described above.

After blue light exposure a reduction in FC was observed in the visual cortices (VIS) and important parts of the DMN, such as in the precuneus/posterior cingulate (PPC) areas, on the other hand in the salience network (SAL) FC increased. The SAL network has been related to the processing of emotional and sensory stimuli^49^ and the medial part of PPC is related to memory^28^. Also, improvement in sleep quality was associated with a decrease in functional connectivity in the SAL network^50^. Hence, while a certain amount of blue light might result in enhanced attention about the environment, the wrong intensity may cause a hypervigilance state, where it is difficult to focus and sustain attention for a long period. Thus, the effects of exposure to blue light seem contradictory, some recent meta-analyses have shown the possible influence on sleep and attention^51^, although some studies have shown increasing attention due to blue-enriched light in the morning^52^. Therefore, further investigations are needed to determine the fine-tuning of where blue light may be beneficial and its link with FC.

Our results shown here are based on individuals who were not challenged with a cognitive task. Moreover, the observation of different FC patterns in each wavelength used in this study were made just after 5 min of stimulation, not during the rs-fMRI. Some interpretation could be made based on our results. In some studies, functional activation of certain cortical regions and task performance has been seen to be influenced by light exposure 30 or 40 min before the task was carried out, suggesting locus coeruleus as principal regulator of alertness and cognition^53^. The results of this study suggest that passive monochromatic light exposure without cognitive or perceptual tasks could lead to changes in brain FC after 5 min of exposure. Studies assessing the relationship between light exposure and brain function have been primarily done with blue light. In some cases, the study of light effects in the brain has been carried out during or immediately after stimulation^20^. Another study found reduced brain activation within the rostral anterior cingulate cortex during an emotional anticipation task 40 min after the end of light exposure^54^. In contrast, brain activation was seen to be enhanced within the dorsolateral and ventrolateral prefrontal cortex during a working memory task 35 min after exposure. In both these studies subjects were exposed to blue light for 30 min. These studies suggest that, at least with blue light, the changes in FC seen in our study are not a signature of a recovery of something occurring during the stimulation, but rather lasting (persistent) effects caused by the light stimulation^53^.

The present study helps to elucidate which would be the most suitable task to capture the putative effects of light exposure on cognition. Concretely, given that the attentional networks (SAL and DAN) as well as the DMN are involved, this suggest the choice of attentional or working memory tasks**.** Future investigations should include task fMRI with different wavelength exposures where the effects on cognitive functions can be specifically tested. Research using light exposure while conducting an fMRI and therefore observing the possible changes in cortical networks associated with visual, attentional, executive and memory functions could elucidate the potential of monochromatic light exposure in health issues, as they become an interesting topic in research^55,56^. A limitation of this pilot study is that we only included a small sample of young individuals. Although we did not find differences between the baseline data***,*** a larger sample including participants of different ages would provide a broader understanding of the results and generalization of the results by controlling the intra-individual variability factor. This study sheds light on how FC organization can be modified by passive light stimulation with only 60 s of exposure and with persisting effects for at least 5 min after this light exposure. Also, our investigation opens new avenues for future studies to better understand how different monochromatic light affects brain function connectivity, for how long stimulation is required, and for how long these effects last.

## Methods

### Eligible participants

Seven participants (N = 7), 4 females and 3 males, were recruited for this pilot study. The mean age at the first fMRI measurement was 28.00 ± 4.50 years (range 21–33 years). The study was approved by the Ethical Committee of Mutua de Terrassa Hospital (Spain), registration number 07/2019, and was carried out according to the Declaration of Helsinki. All participants were informed of the purpose of the study, the nature of the measurements and the possible negative effects. Informed consent was obtained from all subjects and/or their legal guardian(s). To guarantee that candidates were eligible for participation, preliminary testing and a medical examination were done before the start of the study. The preliminary optometric examination consisted of the Ishihara test, best-corrected visual acuity at distance and near, refraction, near point of convergence, stereovision (Randot test), baseline spontaneous blink rate, and pupillary diameter in scotopic and photopic conditions (Table 1). Enrollment was limited to adults between 18 and 35 years old who had not received light exposure therapy within the 6 months before the start of the study.

**Table 1:** Descriptive visual parameters of the participants.

	Refraction	BCVA at 0.4 m (Snellen)	NPC (cm)	SEBR (blink/min)	PD (mm) Mesopic Photopic	ST (secarc)	Color vision
S1	Right = + 0.50 − 0.50 × 120º Left = + 0.75 − 0.50 × 60º	20/20 20/20	6/7	21	6 4	20	Normal
S2	Right = − 0.25 Left = − 0.75	20/20 20/20	6/7	25	6 5	15	Normal
S3	Right = − 0.50 − 0.25 × 180º Left = − 0.25 × 180º	20/20 20/20	10/20	18	7 4	30	Normal
S4	Right = − 3.75 Left = − 3.25–0.50 × 180º	20/20 20/20	6/7	11	6 4	15	Normal
S5	Right = − 0.75 D Left = − 0.75 D	20/20 20/20	6/7	23	7 4	40	Normal
S6	Right = − 0.50 − 0.25 × 100º Left = + 0.25 − 0.25 × 80º	20/20 20/20	5/8	16	7 5	15	Normal
S7	Right = + 1.00 Left = + 0.75 − 0.25 × 90º	20/20 20/20	6/7	18	7 4	20	Normal

*BCVA* Best corrected visual acuity at 40 cm, *NPC* Near point of convergence, first value (break), second value (recovery), *SEBR* Spontaneous eye blink rate, *PD* Pupil diameter, *ST* Stereoacuity.

Subjects with neurological conditions, retinal diseases, color blindness and refractive errors greater than 4D of myopia, 4D of hyperopia and 1D of astigmatism were excluded. In addition, due to fMRI requirements, individuals with cochlear implants, stents, pacemakers, implantable cardioverter defibrillators, shrapnel, metallic fragments, artificial limbs, dental implants, dentures, knee or hip prosthesis, intrauterine devices or any other particle or device containing metal were also excluded. Participants were asked to avoid caffeine, alcohol or any significant changes in diet or sleep patterns 24 h before the experiments. Moreover, a medical examination was conducted by a certified medical doctor to verify that the participants fulfilled all study and scan requirements.

### Study design and experimental setup

**Figure 3:**
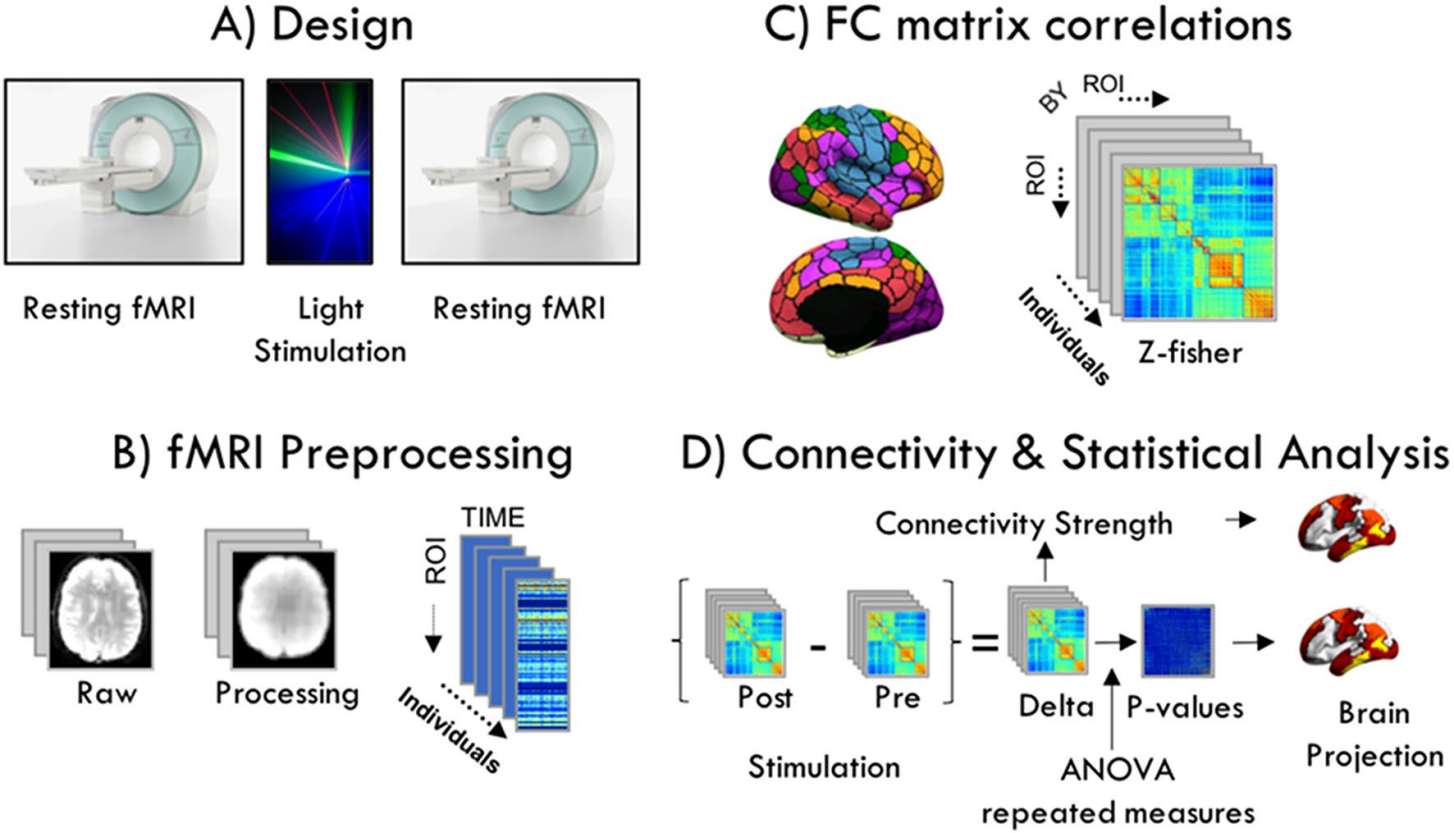
Workflow diagram. (**A**) The experimental design consisted of a repeated measures design where all the subjects underwent a resting fMRI session before and after receiving 1 min of light exposure to one of three wavelengths (green, blue, red) on three different days. (**B**) The fMRI analysis (see details in “Scanning protocol” section) yielded a time series for each individual. (**C**) The functional connectivity matrix for each subject was obtained after performing the Pearson correlation of the individual time series, and then we applied the Z-fisher transformation to perform the statistical analysis. The number of ROIs in the matrix correspond to the 100 ROI atlas used to extract the time series. (**D**) The connectivity analysis of the resting state networks was performed by computing the strength of connectivity within and between each network. To perform the statistical analysis, we first computed the difference between the FC post- and pre-stimulation yielding a delta matrix for each subject. These were used to perform the ANOVA repeated measures to find differences between the functional connections according to the kind of stimulation. This resulted in *p* value matrixes that were subsequently projected into the brain surface.

All participants underwent functional magnetic resonance imaging (fMRI) with their eyes closed before and after 1-min exposure to monochromatic wavelength light. Measurements were done at Fundació Pasqual Maragall facilities in Barcelona, Spain, on three separate days spaced a minimum of one week. A magnetic resonance imaging (MRI) scanner, Philips 3-Tesla (MR Systems Ingenia CX), was used to perform the functional magnetic scans before and after light exposure (Fig. 3).

Upon finalization of the first fMRI subjects were asked to leave the scan room and go to an adjacent room for the light stimulation. Both rooms were kept in dim light and the participants were asked to keep their eyes closed when changing rooms to avoid exposure to ambient light. Once in the adjacent room participants were instructed to put their heads in the Sidtav device. Participants were exposed to one minute of light without corrective lenses (glasses or contact lenses) and with both eyes open. We encouraged them to blink as little as possible without feeling any pain. The viewers were asked to look for one minute directly at the light source through an opening in the device, during this minute the room was kept completely dark. When the minute ended the light-emitting device automatically switched off and the participant went back to the scan room, again with their eyes closed and guided by one of the researchers for the second fMRI. The amount of time between the end of the light stimulation and the start of the fMRI imaging was approximately 5 min.

On each measurement day, all participants used the same device and the same light-color stimulation. The light used on the first measurement day was red, on the second and third days the lights used were green and blue, respectively. The three measurements were spaced apart for a minimum of one week to avoid cross-contamination between light exposures and fMRI measurements. Baseline and light exposure took place on the same day for all the participants with each light color between 9 AM and 2 PM. Approximately 40 min were needed to complete all measurements for each participant.

### Light device

**Figure 4:**
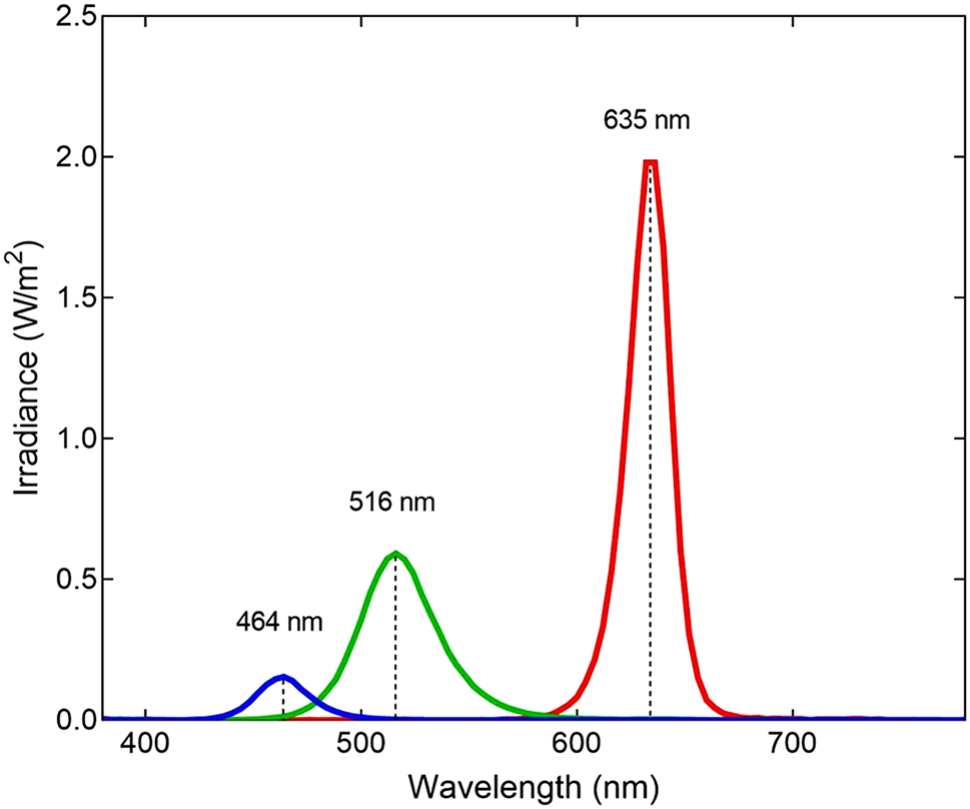
Spectral irradiance (W/m^2^) of the three monochromatic lights used in this study. Irradiance peaks are 464 nm (blue), 516 nm (green) and 635 nm (red).

The light-emitting device used, (Sidtav, Mataró, Spain), is commercially available to conduct monochromatic light exposure in the European Union. It is a hollow rectangular cuboid with a light source on one extreme and an opening on the other. The light source consists of an array of 4 multicolor LED’s at 3.000 K placed at the bottom end of the cuboid, 25 cm away from where the eyes of the subjects sit. The SIDTAV device is connected with a switching adapter with 110–240 V input and 21–27 V output. In front of the light source the device has incorporated a diffusion filter with a transmission of 11% in blue, 7% in green and 5% in red. Diagrams of the instrument and spectral information of the diffusion filter can be found in Extended Data Figure, Figs. S1, S2 and S3. The light emitted by the device can be selected using specifically developed software that can be installed on any computer (Windows). Spectral analysis of the device was made at the Terrassa School of Optics and Optometry (FOOT) which belongs to the Polytechnic University of Catalonia (UPC), using a spectroradiometer (PhotoResearch PR-715, SpectraScan Systems). The three regions of the visible spectrum used in the experiment were analyzed, corresponding to wavelengths near red (635 nm), green (516 nm) and blue (464 nm) (Fig. 4). Photon density for blue, green and red were 1.18·10^19^, 7.24·10^19^ and 1.68·10^20^ photons/m^2^s, respectively. Total Irradiance for blue, green and red were 5.01, 27.7 and 52.7 W/m^2^.

### Scanning protocol

Participants underwent eyes-closed resting-state fMRI using a 12-channel head coil on a Siemens Trio TIM 3 T scanner. A total of 3 imaging rs-fcMRI runs of ~ 6 min each (total 124 volumes) were acquired using the following parameters: 3000 ms, echo time: 30 ms, flip angle: 85°, matrix: 72 × 72, 3 by 3 by 3 mm voxels.

Data were processed using AFNI v20.1 using example 11 as a model. Briefly, each run was slice-time corrected, realigned, normalized (MNI 152 EPI template; Montreal Neurological Institute, Montreal, Canada), and smoothed with a 6 mm FWHM Gaussian kernel. Also, the nuisance signals from the white matter, cerebral spinal fluid and movement parameters (including the first derivatives) were regressed out. We used an eroded mask of white mask and CSF in order to extract the signal. Scrubbing procedures to limit extreme values from hardware instability or head motion^24^ were applied. Specifically, outlier volumes were scrubbed from the connectivity analysis if the change in global signal was greater than 2.5 SDs from the mean change, if the change in position was greater than 0.75 mm from the previous volume, or if the change in rotation was greater than 1.58 degrees from the previous volume. Individual runs were dropped if more than 20 volumes in a run were flagged as outliers, if the mean movement was greater than 0.25 mm, or if the average temporal signal to noise was less than 115.

### Functional connectivity analysis

The time courses signals were extracted from the preprocessed brain images using a spatially continuous and non-overlapping parcellation of 100 ROIs (nodes) released by Schaefer et al.^25^. The nodes correspond to the 7-network parcellation defined by Yeo et al.^26^. Specifically, the visual network (VIS), the auditory and somatosensory network (ASM), the dorsal attention network (DAN), the saliency network (SAL), the orbitofrontal-temporopolar network (ORB), the frontoparietal (FP), and the default mode network (DMN), referred herein to as resting-state networks (RSNs).

The functional connectivity matrices were obtained by computing the Pearson correlation of each node time-courses to every other node time-courses, yielding a 100 × 100 matrix and 4950 connections for each individual. These matrices were Z-fisher transformed to allow statistical comparisons. We computed the differences (deltas) between the post- and pre-stimulation matrices for each kind of light stimulation. Therefore, a delta positive or negative value between any pair of regions indicates that the correlation has increased or decreased after the light exposure, respectively. Then, for each region, we calculated the strength of connectivity of the delta within and between networks using the weighted degree formula, where for a given region (i), its strength is computed by:

$$S\left( i \right) = \mathop \sum \limits_{j = 1}^{n} A_{ij}$$

where *A*_*ij*_ is the ij-th element of the connectivity matrix and *n* is the number of regions (100). This yielded a total of 3 vectors (length 100 × 1) indicating the strength of connectivity of each region after each one of the 3 interventions (light exposure). Afterwards, we computed the Z-score for each vector and projected it onto the brain surface.

We also performed an ANOVA repeated measures with one factor “effect of light exposure”, for each connection on the delta matrices. Specifically, from the 100(ROIs) × 100(ROIs) = 10,000 connections we examined a total of 4950 (100*(99)/2) connections. Thus, a significant *p* value correspond to a given connection between any given pair of ROIs. However, a specific ROI can have multiple significant connections with other ROIs. Thus, each significant *p* value entails a change in the FC (the connections), but also a more sensitive region to light exposure. Thus, a significant *p* value on a given connection would represent the main effect of light stimulation. Then, FDR-correction at 0.05 significance level was applied to control for multiple comparisons^27^. The results were then projected onto a brain surface.

## Supplementary Information

Supplementary Information.

## References

[1] Hamblin MR (2017). Mechanisms and applications of the anti-inflammatory effects of photobiomodulation. AIMS Biophys..

[2] Wahl S (2019). The inner clock-Blue light sets the human rhythm. J. Biophotonics.

[3] Figueiro MG (2016). Delayed sleep phase disorder: Clinical perspective with a focus on light therapy. Nat. Sci. Sleep.

[4] Gold AK, Kinrys G (2019). Treating circadian rhythm disruption in bipolar disorder. Curr. Psychiatry Rep..

[5] Bais B (2016). Bright light therapy in pregnant women with major depressive disorder: Study protocol for a randomized, double-blind, controlled clinical trial. BMC Psychiatry.

[6] Do MT, Yau KW (2010). Intrinsically photosensitive retinal ganglion cells. Physiol. Rev..

[7] Sexton T, Buhr E, Van Gelder RN (2012). Melanopsin and mechanisms of non-visual ocular photoreception. J. Biol. Chem..

[8] Hastings MH, Maywood ES, Brancaccio M (2018). Generation of circadian rhythms in the suprachiasmatic nucleus. Nat. Rev. Neurosci..

[9] West KE (2011). Blue light from light-emitting diodes elicits a dose-dependent suppression of melatonin in humans. J. Appl. Physiol..

[10] Höhn C (2021). Preliminary results: The impact of smartphone use and short-wavelength light during the evening on circadian rhythm, sleep and alertness. Clocks Sleep.

[11] Garcia-Saenz A (2018). Evaluating the association between artificial light-at-night exposure and breast and prostate cancer risk in Spain (MCC-Spain Study). Environ. Health Perspect..

[12] Rodríguez-Morilla B (2018). Blue-enriched light enhances alertness but impairs accurate performance in evening chronotypes driving in the morning. Front. Psychol..

[13] Raikes AC (2020). Daily morning blue light therapy improves daytime sleepiness, sleep quality, and quality of life following a mild traumatic brain injury. J. Head Trauma Rehabil..

[14] Virk G (2009). Short exposure to light treatment improves depression scores in patients with seasonal affective disorder: A brief report. Int. J. Disabil. Hum. Dev..

[15] Connolly LJ (2021). Factors associated with response to pilot home-based light therapy for fatigue following traumatic brain injury and stroke. Front. Neurol..

[16] Newman DP (2016). Ocular exposure to blue-enriched light has an asymmetric influence on neural activity and spatial attention. Sci. Rep..

[17] Noseda R (2016). Migraine photophobia originating in cone-driven retinal pathways. Brain.

[18] Ibrahim MM (2017). Long-lasting antinociceptive effects of green light in acute and chronic pain in rats. Pain.

[19] Figueiro MG (2009). Preliminary evidence that both blue and red light can induce alertness at night. BMC Neurosci..

[20] Vandewalle G (2007). Brain responses to violet, blue, and green monochromatic light exposures in humans: Prominent role of blue light and the brainstem. PLoS ONE.

[21] Ibrahimi D (2021). Changes in the brain activity and visual performance of patients with strabismus and amblyopia after a compete cycle of light therapy. Brain Sci..

[22] Rea MS, Nagare R, Figueiro MG (2021). Relative light sensitivities of four retinal hemi-fields for suppressing the synthesis of melatonin at night. Neurobiol. Sleep Circadian Rhythms.

[23] Peña-Gómez C (2012). Modulation of large-scale brain networks by transcranial direct current stimulation evidenced by resting-state functional MRI. Brain Stimul.

[24] Power JD, Schlaggar BL, Petersen SE (2014). Studying brain organization via spontaneous fMRI signal. Neuron.

[25] Schaefer A (2018). Local-global parcellation of the human cerebral cortex from intrinsic functional connectivity MRI. Cereb. Cortex.

[26] Yeo BT (2011). The organization of the human cerebral cortex estimated by intrinsic functional connectivity. J. Neurophysiol..

[27] Durnez J, Roels SP, Moerkerke B (2014). Multiple testing in fMRI: An empirical case study on the balance between sensitivity, specificity, and stability. Biom. J..

[28] Buckner RL (2009). Cortical hubs revealed by intrinsic functional connectivity: Mapping, assessment of stability, and relation to Alzheimer’s disease. J. Neurosci..

[29] Li Y (2021). Large-scale reconfiguration of connectivity patterns among attentional networks during context-dependent adjustment of cognitive control. Hum. Brain Mapp..

[30] Zhang Z (2021). The longitudinal effect of meditation on resting-state functional connectivity using dynamic arterial spin labeling: A feasibility study. Brain Sci..

[31] Tordjman M (2021). Functional connectivity of the default mode, dorsal attention and fronto-parietal executive control networks in glial tumor patients. J. Neurooncol..

[32] Yu Q (2021). Disorganized resting-state functional connectivity between the dorsal attention network and intrinsic networks in Parkinson’s disease with freezing of gait. Eur. J. Neurosci..

[33] Dai P (2019). Altered spontaneous brain activity of children with unilateral amblyopia: A resting state fMRI study. Neural Plast..

[34] Yang H (2021). Disrupted intrinsic functional brain topology in patients with major depressive disorder. Mol. Psychiatry.

[35] Javaheripour N (2021). Altered resting-state functional connectome in major depressive disorder: A mega-analysis from the PsyMRI consortium. Transl. Psychiatry.

[36] Anticevic A (2010). When less is more: TPJ and default network deactivation during encoding predicts working memory performance. Neuroimage.

[37] Fox MD (2005). The human brain is intrinsically organized into dynamic, anticorrelated functional networks. Proc. Natl. Acad. Sci. U. S. A..

[38] Rajan A (2021). The microstructure of attentional control in the dorsal attention network. J. Cogn. Neurosci..

[39] Suo X (2021). Anatomical and functional coupling between the dorsal and ventral attention networks. Neuroimage.

[40] Liu S (2021). Ocular dominance and functional asymmetry in visual attention networks. Invest. Ophthalmol. Vis. Sci..

[41] Broeders TAA (2021). Dorsal attention network centrality increases during recovery from acute stress exposure. Neuroimage Clin..

[42] Yang S (2021). Enhanced anti-correlation between the dorsal attention and default-mode networks: A resting-state fMRI study of acute insomnia. Neuroscience.

[43] Spadone S (2021). Spectral signature of attentional reorienting in the human brain. Neuroimage.

[44] Gaymard B (1998). Cortical control of saccades. Exp. Brain Res..

[45] Drew AS, van Donkelaar P (2007). The contribution of the human FEF and SEF to smooth pursuit initiation. Cereb. Cortex.

[46] Lee D (2021). Altered functional connectivity of the dorsal attention network among problematic social network users. Addict. Behav..

[47] Wu KR (2020). Altered brain network centrality in patients with adult strabismus with amblyopia: A resting-state functional magnetic resonance imaging (fMRI) study. Med. Sci. Monit..

[48] Wang H (2017). Impaired activation of visual attention network for motion salience is accompanied by reduced functional connectivity between frontal eye fields and visual cortex in strabismic amblyopia. Front. Hum. Neurosci..

[49] Menon V (2011). Large-scale brain networks and psychopathology: A unifying triple network model. Trends Cogn. Sci..

[50] Ma J (2020). Decreased functional connectivity within the salience network after two-week morning bright light exposure in individuals with sleep disturbances: A preliminary randomized controlled trial. Sleep Med..

[51] Srisurapanont K (2021). Blue-wavelength light therapy for post-traumatic brain injury sleepiness, sleep disturbance, depression, and fatigue: A systematic review and network meta-analysis. PLoS ONE.

[52] Studer P (2019). Effects of blue- and red-enriched light on attention and sleep in typically developing adolescents. Physiol. Behav..

[53] Killgore WDS (2020). Blue light exposure enhances neural efficiency of the task positive network during cognitive interference task. Neurosci. Lett..

[54] Alkozei A, Smith R, Killgore WD (2016). Exposure to blue wavelength light modulates anterior cingulate cortex activation in response to ‘uncertain’ versus ‘certain’ anticipation of positive stimuli. Neurosci. Lett..

[55] Terman M (2007). Evolving applications of light therapy. Sleep Med. Rev..

[56] Hanford N, Figueiro M (2013). Light therapy and Alzheimer's disease and related dementia: Past, present, and future. J. Alzheimers Dis..

