# Supplementary material for: Functional connectivity of brain networks with three monochromatic wavelengths: a pilot study using resting-state functional magnetic resonance imaging
Source: Sci Rep. 2022 Sep 28;12:16197. doi: 10.1038/s41598-022-20668-9 (PMC9519584; doi:10.1038/s41598-022-20668-9)

Extended Data Figures

Fig S1. Specifications of the instrument used for this study (Sidtav, Barcelona, Spain). The light source is an array of 4 multicolor 3.000K LED's. A 3.5 cm diameter diffusion filter is placed in front of the light source. Distance from the light source to the opening where the observer places its eyes is 25.0 cm.

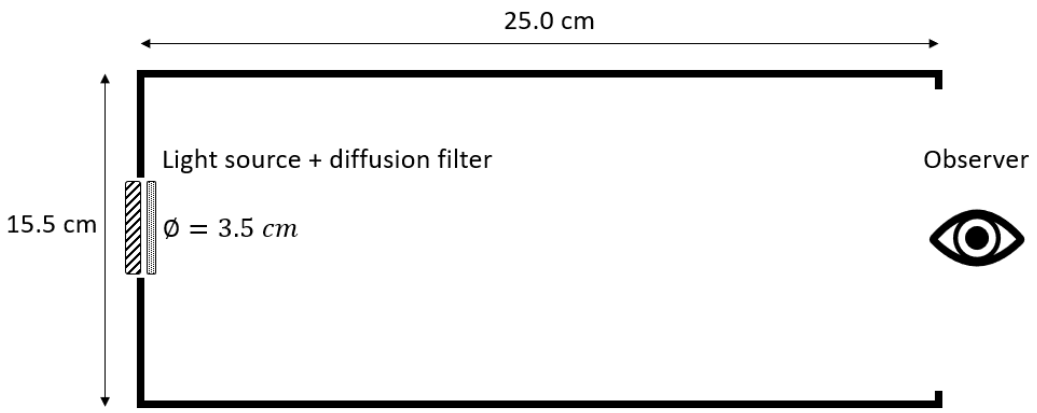

Fig S2. Picture from the settings used to measure the spectrometry for the three monochromatic lights used in this study. Spectral analysis of the device was made with the spectroradiometer PhotoResearch PR-715, SpectraScan Systems.

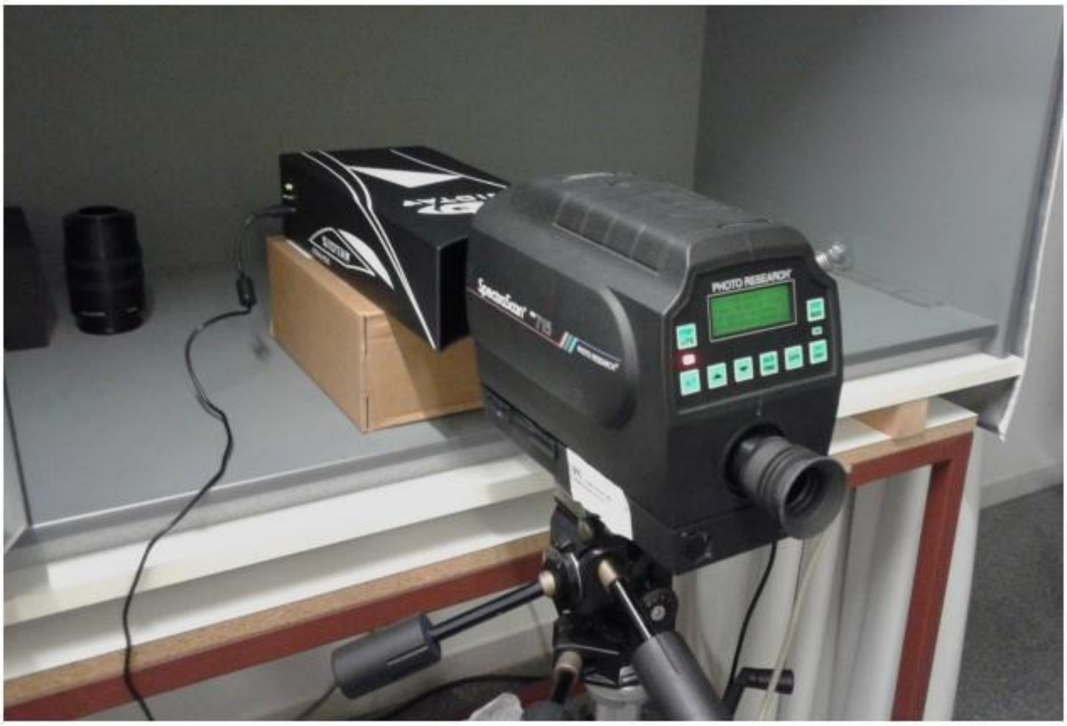

Fig S3. Normalized spectral transmittance of the diffusion filter of the device used in this study. We extracted the filter from the instrument and placed it in front of a white smartphone screen at the highest contrast. We first measured the screen directly at the spectroradiometer and then with the diffusion filter on the screen. Transmission was 11% in blue, 7 % in green and 5 % in red.

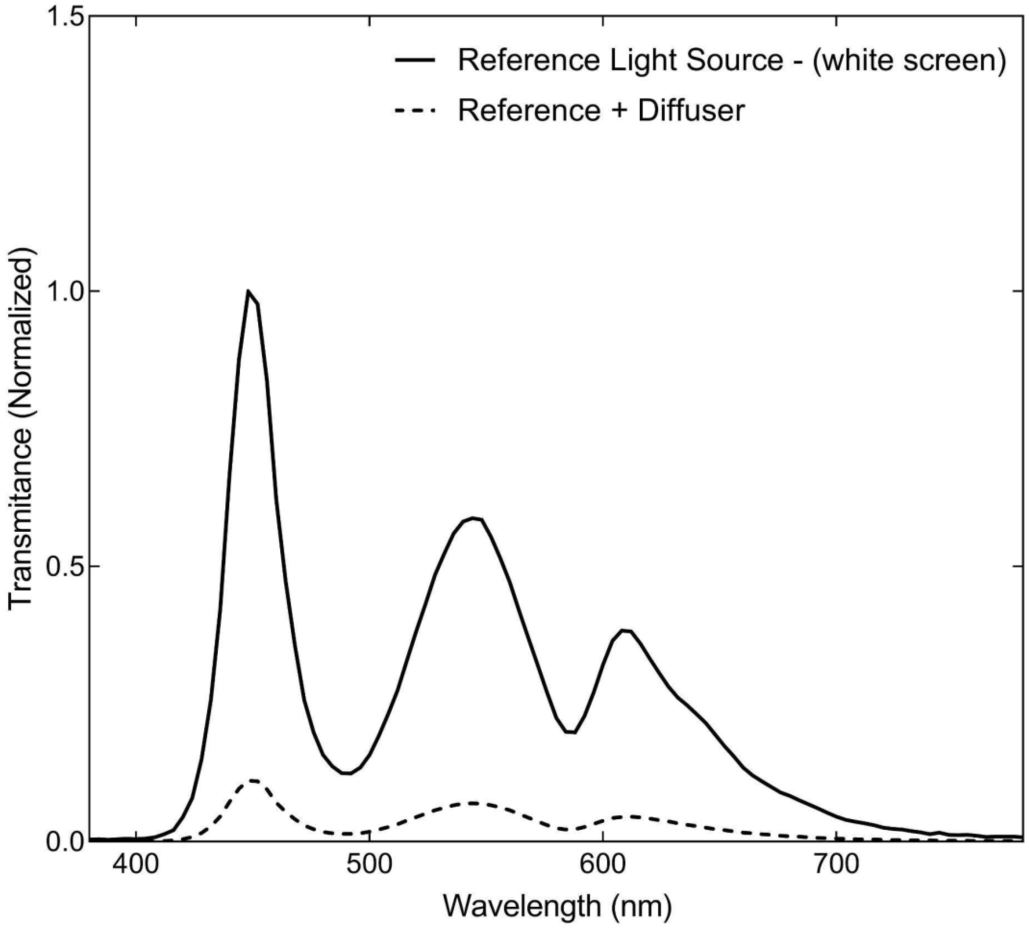

Supplement: Supplementary file 1 — Supplementary Information. [file 41598_2022_20668_MOESM1_ESM.pdf]
